# Harnessing generative AI to decode enzyme catalysis and evolution for enhanced engineering

**DOI:** 10.1093/nsr/nwad331

**Published:** 2023-12-28

**Authors:** Wen Jun Xie, Arieh Warshel

**Affiliations:** Department of Medicinal Chemistry, Center for Natural Products, Drug Discovery and Development, Genetics Institute, University of Florida, Gainesville, FL 32610, USA; Department of Chemistry, University of Southern California, Los Angeles, CA 90089, USA

**Keywords:** generative AI, mutation effects, evolution–catalysis relationship, enzyme engineering, enzyme evolution

## Abstract

Enzymes, as paramount protein catalysts, occupy a central role in fostering remarkable progress across numerous fields. However, the intricacy of sequence-function relationships continues to obscure our grasp of enzyme behaviors and curtails our capabilities in rational enzyme engineering. Generative artificial intelligence (AI), known for its proficiency in handling intricate data distributions, holds the potential to offer novel perspectives in enzyme research. Generative models could discern elusive patterns within the vast sequence space and uncover new functional enzyme sequences. This review highlights the recent advancements in employing generative AI for enzyme sequence analysis. We delve into the impact of generative AI in predicting mutation effects on enzyme fitness, catalytic activity and stability, rationalizing the laboratory evolution of *de novo* enzymes, and decoding protein sequence semantics and their application in enzyme engineering. Notably, the prediction of catalytic activity and stability of enzymes using natural protein sequences serves as a vital link, indicating how enzyme catalysis shapes enzyme evolution. Overall, we foresee that the integration of generative AI into enzyme studies will remarkably enhance our knowledge of enzymes and expedite the creation of superior biocatalysts.

## Teaser

This Review underscores the latest advances in applying generative AI to the analysis of enzyme sequences, which has deepened our insight into enzyme evolution and catalysis, thereby advancing enzyme engineering.

## INTRODUCTION

Enzymes serve as biological catalysts that expedite cellular chemical reactions without being depleted [1]. They are essential in various processes, including metabolism, digestion, gene regulation and cellular signaling. The significance of enzymes extends into biotechnology, medicine and industry, where they find applications in the production of pharmaceuticals, biofuels, diagnostics and targeted treatments [2,3]. Thus, improving our knowledge of enzymes and making advancements in enzyme engineering is essential [4–7].

Numerous studies have focused on enzymes, particularly exploring their structure, function and mechanism [8–11]. The conventional experimental approaches in use include enzyme kinetics analysis, X-ray crystallography, nuclear magnetic resonance spectroscopy and cryo-electron microscopy. A bottleneck in these experimental methods, however, lies in their low throughput, which may be augmented by computational solutions. Computational methods like molecular dynamics simulations and quantum mechanics/molecular mechanics for free energy calculations offer insights that might be otherwise challenging or highly resource intensive with experimental approaches. However, a comprehensive and predictive understanding of enzyme behavior still presents a significant challenge.

Machine learning has recently demonstrated its ability to bolster both the analysis and engineering of proteins, including enzymes (as discussed in refs [12–26]). Machine learning algorithms are broadly classified into two groups: discriminative models and generative models. Discriminative models specialize in data classification or labeling. In contrast, generative AI seeks to identify the innate distribution of data, thereby enabling the generation of new instances informed by this distribution. When implemented with enzyme sequences, generative models reveal latent patterns and tendencies in the immense sequence space, ultimately aiding in the identification of novel functional sequences. Several reviews have summarized the application of generative models to proteins from an engineering perspective [19,26]. There is a pressing need for an exploration aimed at deciphering proteins, particularly enzymes, in a way that resonates with biophysicists’ perspective, as they seek to decode the physical principles behind the structure and function of enzymes, guiding a more rational approach to enzyme engineering. This is the main objective of the present review.

In this review, we examine the latest progress in applying generative AI to enzymes, focusing on the analysis of natural protein sequences. We begin with an overview of several notable generative models. Next, we discuss the progress made by generative models in deepening our insight into enzyme evolution, architecture and function. In particular, generative models analyzing natural enzyme sequences could yield a metric that correlates with enzyme fitness, catalytic activity and stability, thereby indicating how the physicochemical properties of enzymes shape their evolution. Even for *de novo* enzymes utilizing enzyme scaffolds from nature, natural evolutionary information proves to be insightful to rationalize their laboratory evolution. We then explore the application of generative models for enzyme engineering. Lastly, we discuss the challenges and future prospects of employing generative models in enzyme studies. We envision that generative AI has the potential to resolve persistent issues in enzyme research and sculpt intelligent strategies for enzyme engineering.

## AN OVERVIEW OF GENERATIVE MODELS

Generative models are a category of machine-learning approach that uncovers the fundamental distribution of a data set, enabling the creation of new samples that follow this distribution [27]. This generative capability proves particularly advantageous for data synthesis tasks, such as generating text, images or sounds resembling the original training data. Given the abundance of homologous sequences from various organisms, the application of generative models to enzyme design is a natural fit [14,18,19,22,25], as the sequence probability learned may also carry biological and evolutionary implications. In this setting, we provide a succinct introduction to several generative models pertinent to enzyme sequence studies, including maximum-entropy (MaxEnt) models [28], variational autoencoders (VAEs) [29], language models [30] and generative adversarial networks (GANs) [31].

While all these diverse models fall under the umbrella of generative AI, each offers a distinct suite of characteristics, including distinct formulations, interpretative capacities and generative competencies, as summarized in Table 1. Frameworks such as MaxEnt models, VAEs and language models explicitly furnish sequence probabilities; in contrast, GANs are not typically classified within the standard probabilistic model framework. MaxEnt models provide a more intuitive interpretation despite not explicitly addressing higher-order residue interactions as other models do. Setting them apart, language models eliminate the need for multiple sequence alignment (MSA) during training, a quality that allows them to conduct research across diverse protein families within a single model. These nuances suggest the potential for certain models to surpass others in specific tasks, highlighting the significance of deliberate model selection in machine-learning research.

**Table 1.** Features of representative generative AI applied in protein sequence analysis.

**Model type**	**Residue coupling**	**Sequence probability**	**MSA independent**	**Interpretable parameter**	**Alternative name**
MaxEnt	Pairwise	✓	×	✓	DCA, EVcoupling, GREMLIN, etc.
VAE	Higher-order	✓	×	×	DeepSequence
Language	Higher-order	✓	✓	×	ESM
GAN	Higher-order	×	×	×	

### Maximum entropy model

**Figure 1.**
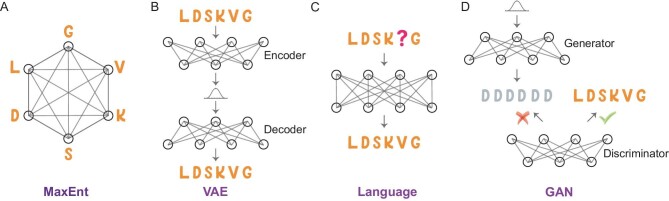
Comparative illustration of generative models utilized for protein sequence modeling. (A) MaxEnt model: This model aims to delineate both the conservation of individual amino acids and their pairwise interactions, while concurrently making minimal assumptions by maximizing sequence information entropy. (B) VAE: A neural network that learns to encode data into a lower-dimensional latent space and then decode it back; after training, it can effectively generate new data that resemble the training set. (C) Language model: A masked language model employs a prediction-based mechanism that strives to accurately forecast the masked amino acid, thus learning the distribution of a corpus of protein sequences. (D) GAN: A framework utilizes two neural networks operating in tandem—a generator that creates new protein sequences, and a discriminator that evaluates them for authenticity.

MaxEnt models constitute a specific class of probabilistic models that calculate a data set's probability distribution while maximizing information entropy given a set of constraints derived from the observed data [28]. These models are rooted in information theory and statistical mechanics. In analyzing protein sequences, MaxEnt models usually take into consideration evolutionary conservation and pairwise residue correlation derived from MSA (Fig. 1A). The pairwise residue correlation mirrors epistasis, wherein the impact of mutations on one residue is influenced by another residue, possibly due to residue interactions or functional associations. By integrating these constraints, MaxEnt models provide a framework for capturing the intricate dependencies and patterns present within protein sequences. The MaxEnt model, which serves as a generic framework, has been referred to as a Potts model, Boltzmann machine [32], direct coupling analysis (DCA) [33], EVcoupling [34], GREMLIN [35] and CCMpred [36], among other names in protein studies.

MaxEnt models use the formula $P( {\boldsymbol{S}} ) \propto exp( { - E( {\boldsymbol{S}} )} )$ to compute the probability $P( {\boldsymbol{S}} )$ for a sequence ${\boldsymbol{S}}$, where $E( {\boldsymbol{S}} ) = \mathop \sum \nolimits_i {h}_i{S}_i + \mathop \sum \nolimits_{i > j} {J}_{ij}{S}_i{S}_j$ represents the sequence statistical energy. The model parameters ${h}_i$ and ${J}_{ij}$ can be effectively trained using gradient-based optimization techniques. Depending on the conventions used, some studies might represent $E( {\boldsymbol{S}} )$ with a negative sign, while others might not.

### Variational autoencoder

VAEs are generative models that blend probabilistic modeling with deep neural-network-based encoders and decoders [29]. VAEs operate on the principle of mapping input data to a low-dimensional latent space. Once the encoder has successfully mapped the input data to the latent space, the decoder learns to reconstruct the original input data from this space (Fig. 1B). The latent space provides a representation of the data's structures. A key advantage of VAEs over traditional autoencoders is their generative ability. To achieve this, the decoder generates new data points using samples taken from the latent space. A notable instance of VAE being used on protein fitness prediction is DeepSequence [37].

The log probability of a protein sequence ${\boldsymbol{S}}$, $\log P( {\boldsymbol{S}} )$, can be approximated using the evidence lower bound (ELBO) ${E}_q[ {\log p( {{\boldsymbol{S}}{\mathrm{|}}{\boldsymbol{z}}} )} ] - {D}_{KL}[q({\boldsymbol{z}}|{\boldsymbol{S}})||p( {\boldsymbol{z}} )]$ [29]. In this expression, $q({\boldsymbol{z}}|{\boldsymbol{S}})$ and $p({\boldsymbol{S}}|{\boldsymbol{z}})$ represent neural networks responsible for encoding protein sequence data into latent variables and decoding from the latent space to reconstruct the original sequence data, respectively. The latent variables’ prior distribution, $p( {\boldsymbol{z}} )$, is commonly represented by a Gaussian normal distribution; the Kullback-Leibler (KL) divergence is a measure of the difference between two probability distributions.

### Language model

Language models used in natural language processing determine the probability distribution of a sequence of words by assigning probabilities to each potential word combination, reflecting their likelihood of occurrence within the language [30]. Similarly, protein sequences can be treated as a form of language where amino acids replace words in natural languages as tokens. When tailored appropriately, protein language models can capture the distribution, patterns and relationships observed within protein sequence data, enabling them to generate novel sequences and predict protein attributes. ESM is a widely used pre-trained language model for proteins [38].

Deep-learning techniques, such as recurrent neural networks (RNNs), transformers and autoregressive models, can be used to train protein language models. Both RNNs and transformers can be trained using masked language models, which predict a hidden amino acid based on the context provided by adjacent amino acids in the sequence (Fig. 1C). The probability of a protein sequence ${\boldsymbol{S}}$ is given by $P( {\boldsymbol{S}} ) = \mathop \prod \nolimits_i p({S}_i|{S}_{ - i})$. In the case of autoregressive models, they are employed to model protein sequences by learning the probability distribution of amino acids in a sequence, with each amino acid conditioned on its predecessors. The probability of a protein sequence ${\boldsymbol{S}}$ can be computed using the chain rule of probability $P( {\boldsymbol{S}} ) = \mathop \prod \nolimits_i p({S}_i|{S}_1, \ldots ,{S}_{i - 1})$.

### Generative adversarial network

GANs comprise two distinct neural networks: the generator and the discriminator (Fig. 1D) [31]. The generator network takes random noise as input and produces new data points intended to mimic the training data. Meanwhile, the discriminator network uses both the training data and generated data as inputs to differentiate them. The training process involves refining the generator network to create data points that can deceive the discriminator network, while simultaneously improving the discriminator network's ability to accurately discern between real and fake data points. Therefore, when used in analyzing protein sequences, GANs may distinguish real protein sequences and sequences that do not follow the rules that proteins should have.

In the realm of protein studies, GANs can differentiate authentic protein sequences from those not adhering to conventional protein structures. However, unlike other generative models, GANs do not present an explicit probability distribution for their samples. For tasks that demand an estimation of sequence probability distribution, which we will delve into further below, one might lean towards models like MaxEnt models, VAEs or language models.

## PREDICTING MUTATION EFFECTS ON ENZYME FITNESS

Generative models can assign a probability to any specific enzyme sequence based on the model's comprehension of the inherent distribution within natural protein sequence data. It is intriguing to explore the factors that contribute to the prevalence of specific sequences or variants in nature. To this end, it is essential to bridge the connection between the probability of a certain enzyme sequence in natural protein repositories and its phenotype, which can be assessed in different experiments.

**Figure 2.**
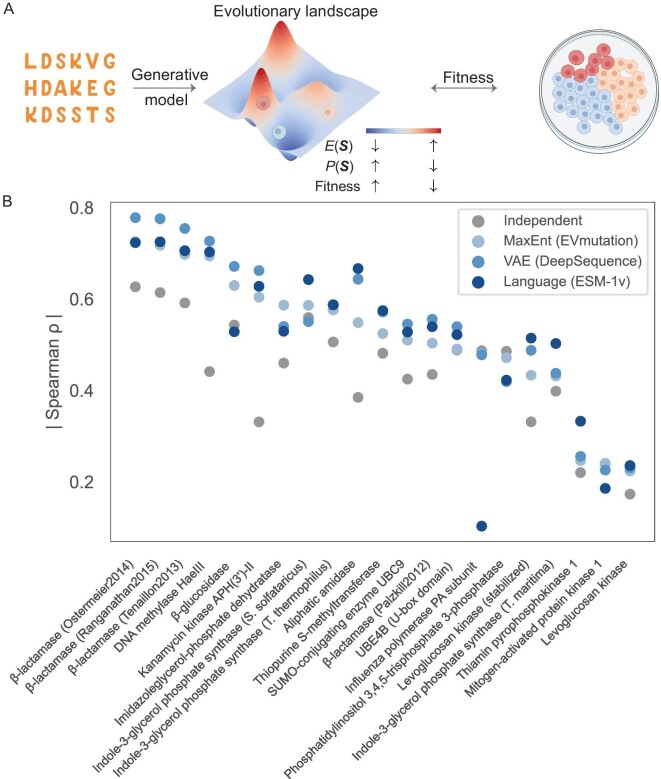
Exploration of enzyme fitness through generative models. (A) Generative models trained on protein sequence data produce a probability distribution that correlates with protein fitness, as determined in DMS. While the evolutionary landscape is intrinsically high-dimensional, we offer a schematic illustration for explanation. Cells in the plate are illustrated using BioRender. (B) Strong correlations are evident across various generative models for a range of enzymes, as highlighted by the Spearman's correlation $\rho $ between sequence probability and fitness. The illustration incorporates several models, including the MaxEnt model, a VAE and a language model, as represented by EVmutation [25], DeepSequence [37] and ESM-1v [38], respectively. (The data used to generate this figure were sourced from ref. [38], focusing exclusively on enzymes.)

Deep mutational scanning (DMS) offers a high-throughput approach for measuring the functional impact of various protein variants in a single experiment, by creating a large library of protein mutants and examining their effects on a specific phenotype [39]. Given that enzymes play a pivotal role in numerous biological pathways, the growth rate of a species could serve as a phenotype to gauge the impact of mutations. Meanwhile, generative models offer an evolutionary landscape to quantify the sequence probability of each mutant. Hence, drawing correlations between sequence probability quantified by the generative model and DMS outcomes can aid in unearthing the biological implications embedded within generative models (Fig. 2A).

To achieve this, several generative models have been deployed across diverse enzymes, revealing a significant correlation between sequence probability in generative models and mutant fitness (Fig. 2B). Overall, VAE and language models tend to excel beyond the MaxEnt model, though each surpasses the independent model. Notably, their performance is not uniform across all enzymes. For certain enzymes, the correlations are not significant, potentially due to DMS experimental noise or the intricacies of evolution. In the following, we will showcase the differing performances of various models.

To our knowledge, the MaxEnt model was the first to be used to associate its sequence statistical energy, or sequence probability, with outcomes of the DMS experiment. Figliuzzi *et al.* used DCA to investigate beta-lactamase TEM-1, an enzyme conferring resistance to beta-lactam antibiotics [40]. They discovered a significant correlation between the statistical energy $E( {\boldsymbol{S}} )$ (i.e. $ - {\mathrm{log}}P( {\boldsymbol{S}} )$) and the minimum inhibitory concentration of the antibiotic, which assesses the enzyme's fitness in promoting survival in antibiotic environments. In a more extensive analysis, Hopf *et al.* corroborated that this relationship prevails across a diverse array of enzymes and other proteins using EVcoupling [25]. This correlation surpasses other metrics, such as the position-specific scoring matrix (PSSM) or the conservation score derived from the MSA profile, highlighting the role of epistasis in shaping protein function.

It is still noteworthy to acknowledge that the PSSM often serves as a suitable initial approach (Fig. 2B). The introduction of epistasis appears to outperform the PSSM, yet in numerous instances, the enhancement brought about by the epistasis model is modest. This could be attributed to the dominance of single mutations in the DMS data set. Although coevolutionary information stemming from the epistasis between two residues is vital for protein structure prediction [41], residue conservation seems to play a predominant role in predicting protein fitness in the current data set. This aligns with the observation that, predominantly, the cumulative changes in free energy of reaction resulting from single mutations closely approximate the free energy alteration measured in the multi-mutant across several proteins [42].

While the MaxEnt model usually considers second-order epistasis, explicitly incorporating higher-order interactions is difficult due to the vast number of model parameters involved. In contrast, VAEs inherently incorporate higher-order correlations. It would be intriguing to investigate the significance of higher-order epistasis in predicting fitness. Riesselman *et al.* employed DeepSequence, a VAE framework, to relate sequence probability (estimated as ELBO) to fitness scores, resulting in slightly enhanced correlation values compared to the epistatic MaxEnt model for a data set encompassing numerous enzymes and other proteins (Fig. 2B) [37].

MaxEnt models and VAEs necessitate the creation of aligned MSA. In contrast, language models offer an added degree of flexibility, capable of employing unaligned protein sequences as input. This trait permits the inclusion of a variety of protein classes into the language model. Language models have been deployed to predict the fitness of protein variants, a significant proportion of which are enzymes, demonstrating comparable correlation values to other models. For instance, ESM-1v [38], trained with masked language models on the UniRef90 database (98 million sequences), demonstrates an impressive capacity to predict mutation effects on enzyme fitness, marginally outperforming both the MaxEnt model and VAE (Fig. 2B).

Despite the comprehensive data set generated by DMS, it primarily focuses on a single functional readout, which could not capture the full complexity of a protein's sequence-function relationship [43]. Consider the widely recognized DMS data set of beta-lactamase TEM-1 provided by Firnberg *et al.* [44]. Synonymous mutations may lead to discernible variations in fitness values, gauged by the minimum inhibitory concentration of the antibiotic, complicating the correlation between fitness values and changes in protein sequences. Furthermore, numerous factors influence an enzyme's fitness, such as enzyme activity and protein abundance, which could be affected by protein stability. Associating fitness with the catalytic activity and stability of enzymes remains challenging. Thus, while generative models can predict fitness, there remains a considerable gap in using such models to further predict enzyme physicochemical properties.

In addition to the zero-shot fitness prediction illustrated above in this section, the hidden states of RNNs, an alternative approach to training language models, can be used to train supervised models for many downstream applications. For example, Alley *et al.* employed an RNN with the UniRef50 database (24 million sequences) as input, finding that the RNN's hidden state could effectively predict protein stability and fitness data [45]. Rives *et al.* also applied a transformer model to analyze the UniParc database (250 million sequences) and predict mutation effects on fitness [46]. Besides, there are many noteworthy studies focused on learning protein representations that can be fine-tuned for diverse tasks [47–49].

## PREDICTING MUTATION EFFECTS ON ENZYME ACTIVITY AND STABILITY

Assessing catalytic activity and stability of enzymes is crucial for understanding biochemical mechanisms, optimizing bioindustrial applications, guiding therapeutic enzyme interventions and ensuring the efficacy of enzyme-based products; however, predicting the influence of mutations on such physicochemical attributes of enzymes has proven to be particularly arduous. Compared to protein thermostability prediction [50], forecasting enzyme activity poses a more substantial challenge. It frequently necessitates multiscale simulations for modeling chemical reactions, which consume significant computational resources [4,8]. For certain mutations, the difference from the wild-type could be relatively small; for instance, a 10-fold rate difference translates to a reaction barrier difference of just 1.4 kcal/mol. Such a negligible difference might exceed the precision of available physical models. Meanwhile, some mutations might provoke large conformational changes [51], which add to the already convoluted task of modeling enzyme catalysis.

Aside from physics-based models, predicting the effects of mutations on enzyme activity using supervised learning is currently unfeasible, largely because of the limited data on enzyme turnover numbers [52]. Although millions of enzyme mutations have been measured and cataloged in databases such as BRENDA and SABIO [53,54], many entries either lack information on experimental conditions or were measured under varying conditions in different laboratories, thus complicating direct comparisons. It is well acknowledged that experimental factors like pH and temperature could considerably impact enzyme catalysis. Thus, predicting the impact of mutations on enzyme activity continues to be both a formidable and vital undertaking that demands innovative strategies.

One might question if a connection exists between sequence probabilities in generative models and enzyme properties such as catalytic activity and thermostability. Identifying a reliable link could lead to predictions of these physicochemical traits in a manner analogous to fitness predictions. Yet, it is crucial to remember that factors determining organism fitness often interrelate. The complex interplay between enzyme catalytic activity (${k}_{cat}$) and protein thermostability serves as a prime example [55–59]. In light of these complexities, drawing a direct association between sequence probability and individual enzyme properties can be intricate.

**Figure 3.**
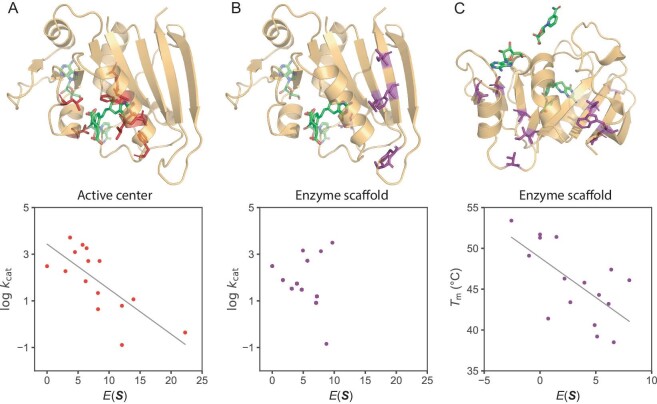
Strong correlation between enzyme physicochemical properties and evolutionary information as extracted from sequence data using generative AI, showcased using DHFR. (A) The $E( {\boldsymbol{S}} )$ (i.e. $ - {\mathrm{log}}P( {\boldsymbol{S}} )$) exhibits a correlation with enzyme activity (${k}_{cat}$) for mutations in the vicinity of the substrate or cofactor, indicated by a Pearson correlation coefficient of −0.74. Mutated sites within the data set are highlighted. (B) The $E( {\boldsymbol{S}} )$ does not show any correlation with enzyme activity for mutations located on the enzyme scaffold. Mutated sites within the data set are denoted. (C) For mutations occurring on the enzyme scaffold, the $E( {\boldsymbol{S}} )$ displays a correlation with thermostability (${T}_{\mathrm{m}}$), reflected by a Pearson correlation value of −0.65. Mutated sites are also marked in purple. (The data used to generate this figure were sourced from ref. [60].)

Nevertheless, using the MaxEnt model, we scrutinized 12 representative enzyme-substrate pairs frequently examined in computational chemistry studies, and uncovered a link between $E( {\boldsymbol{S}} )$ (i.e. $ - {\mathrm{log}}P( {\boldsymbol{S}} )$) and enzyme properties [60]. The crux of our approach was the separate consideration of distinct enzyme regions. An illustration of the relationship is depicted in Fig. 3 for the enzyme dihydrofolate reductase (DHFR). When mutations occur on residues proximal to the substrate or the active center, their sequence probabilities were found to align with enzyme ${k}_{cat}$ values (Fig. 3A). In contrast, mutations on the enzyme scaffold, distant from the substrate, exhibited sequence probabilities that aligned more with enzyme thermostability (${T}_{\mathrm{m}}$) than with ${k}_{cat}$ (Fig. 3B–C). Beyond DHFR, this observation appears consistent across several enzymes that catalyze different types of chemical reactions [60].

It is known that structural and functional constraints influence sequence variation rates at diverse sites [61,62], and the study of patterns within extant sequences offers valuable perspectives on protein functionality [63]. Here the generative model serves as a structured framework for extracting otherwise elusive evolutionary information from the high-dimensional sequence landscape, which can be correlated with enzyme physicochemical properties.

**Figure 4.**
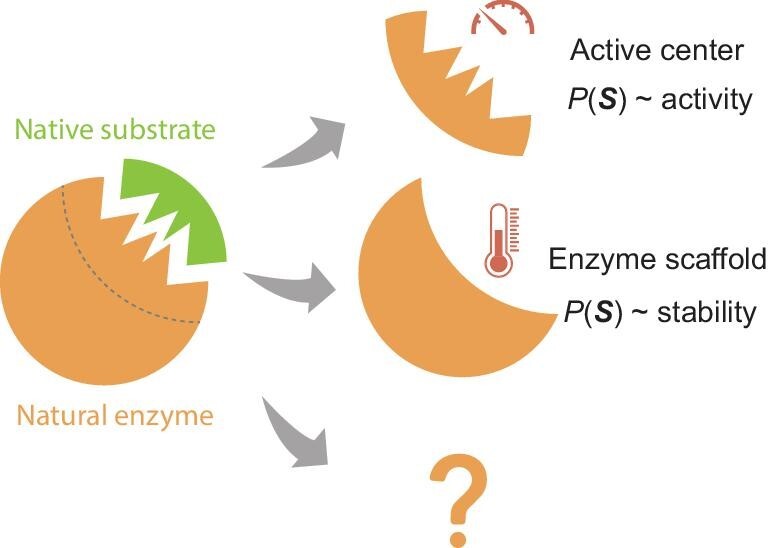
The evolution-catalysis relationship for enzymes. The sequence probability, $P( S )$, derived from generative models, correlates with enzyme activity when mutations occur at the active center and with stability when mutations are in the scaffold region. Further examination of more evolutionary pressures and enzyme regions might reveal new classifications or fine-tune the existing ones.

We term the identified correlations the ‘evolution-catalysis relationship’, linking evolutionary information to factors that impact enzyme catalysis, including catalytic activity and stability (Fig. 4). The relationship implies that nature mainly utilizes different regions on enzymes to enhance different properties, thereby optimizing overall enzyme catalysis. Thus, the relationship provides a way to understand enzyme functional architecture. This comprehension becomes increasingly significant as it produces testable hypotheses. The most straightforward application pertains to suggesting mutations to enhance specific enzyme properties, as demonstrated successfully in ref. [64] (also see discussions in the section ‘Applications in enzyme engineering’). However, given the vast diversity of enzymes, as demonstrated by the wide array of chemical reactions they catalyze, it is possible that further study of enzymes or specific enzyme regions may reveal additional categories in terms of functional architecture [65].

A fundamental question in enzyme evolution revolves around whether natural enzymes are evolving towards higher ${k}_{cat}$ or ${k}_{cat}/{K}_{\mathrm{M}}$ values. While on one side, it intuitively seems that a more proficient enzyme would save an organism's energy, it is also evident that the catalytic efficiency of many enzymes is not optimal. This paradox remains an unresolved issue, as extensively discussed by Milo and Tawfik [66,67]. However, the evident link between sequence probability in generative models and both ${k}_{cat}$ and ${k}_{cat}/{K}_{\mathrm{M}}$, as depicted in Fig. 3A and ref. [60], implies a trend where many enzymes might indeed be on a trajectory towards enhanced activity during natural evolution. Besides, the observed correlation between evolutionary information and enzyme stability in the scaffold region aligns with studies on non-enzyme proteins [68], where enhancing stability could be the dominant evolutionary pressure.

## RATIONALIZING THE LABORATORY EVOLUTION OF *DE NOVO* ENZYMES

**Figure 5.**
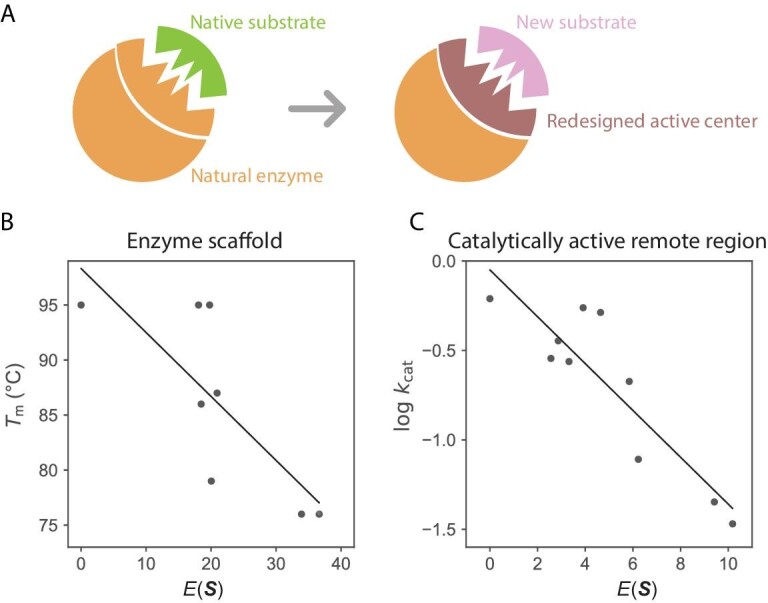
Insights from the MaxEnt model applied to *de novo* Kemp eliminase. (A) Kemp eliminase adopts the TIM-barrel scaffold of a natural synthase; its active center is modified to accommodate a new substrate. (B) Mutations on the Kemp eliminase scaffold show a correlation between $E( {\boldsymbol{S}} )$ and ${T}_{\mathrm{m}}$, evidenced by a Pearson correlation value of −0.77. (C) Mutations in the catalytically active remote region correlate their $E( {\boldsymbol{S}} )$ values with ${k}_{cat}$, indicated by a Pearson correlation value of −0.89. (The data used to generate this figure were sourced from ref. [65].)

We expanded our research to include *de novo* enzymes, specifically focusing on Kemp eliminases, using generative models [65]. In nature, there are no recognized enzymes that can catalyze Kemp elimination [69], which initially poses a challenge in leveraging natural sequence data to analyze Kemp eliminases. However, we discovered that the scaffold of the Kemp eliminase, which originates from an imidazole glycerol phosphate synthase with a triosephosphate isomerase (TIM)-barrel scaffold, provides crucial insights into the function of this *de novo* enzyme (Fig. 5A).

We trained the MaxEnt model using homologs of the natural synthase. The mutations from directed evolution of Kemp eliminase are predominantly located on the synthase scaffold; we assign each mutant $E( {\boldsymbol{S}} )$ (i.e. $ - {\mathrm{log}}P( {\boldsymbol{S}} )$). Notably, the statistical energy $E( {\boldsymbol{S}} )$ correlates with the thermostability of the Kemp eliminase variants (Fig. 5B), although the model is trained with natural synthase homologs [65]. This finding aligns with our observations regarding natural enzymes, where evolutionary information of the enzyme scaffold mainly correlates with enzyme stability [60].

There is another data set that utilized molecular dynamics simulations and biochemistry experiments to pinpoint regions in Kemp eliminase with dynamics coupled to the active site [70]. The majority of these mutations were situated on the TIM-barrel loops, which we have designated as the ‘catalytically active remote region’. Intriguingly, $E( {\boldsymbol{S}} )$ of mutations in this region exhibit a strong correlation with enzyme catalytic activity, which markedly differs from other sites on the enzyme scaffold (Fig. 5C) [65]. A possible molecular mechanism could involve substrate-gated loop rearrangement determining enzyme activity, as others have posited [71]. Once the substrate enters the pocket, loop rearrangement excludes water molecules from the pocket, subsequently enhancing catalytic activity. This reasoning suggests that the role of some TIM-barrel loops is not overly sensitive to a specific chemical reaction, a hypothesis that could be investigated in future experiments.

The findings here for Kemp eliminases suggest that traits from naturally evolved enzyme scaffolds can be integrated into novel functions, which might help to illuminate the origins of new enzymatic activities. This might also explain the prominence of the TIM-barrel scaffold in ∼10% of all enzymes.

Altogether, our studies of enzymes, encompassing both natural and *de novo* enzymes, bring fresh insights into enzyme architecture, catalysis and evolution, giving rise to several hypotheses that warrant further investigation through biochemistry experiments and computational chemistry studies. Meanwhile, we recognize that the correlations depicted in Fig. 3 and detailed in refs [60] and [65] are not perfect, which is anticipated given the intertwined factors influencing enzyme evolution. Delving deeper into various evolutionary pressures and examining different enzyme regions will be crucial to further elucidate and understand the evolution-catalysis relationship.

## DECODING PROTEIN SEQUENCE SEMANTICS

Generative models excel not only in predicting the consequences of mutations but also in discerning the inherent semantics and biological structures ingrained in protein sequences. The term ‘semantics’ refers to the meaningful patterns or relationships that exist within the data—in this case, the functional and structural significance encoded in protein sequences. Here, we present several insightful studies that harness generative models to enhance our comprehension of proteins. While these investigations do not explicitly focus on enzymes, nor have they been applied to enzyme studies yet, they provide adaptable strategies that could be customized for enzyme research in forthcoming pursuits.

**Figure 6.**
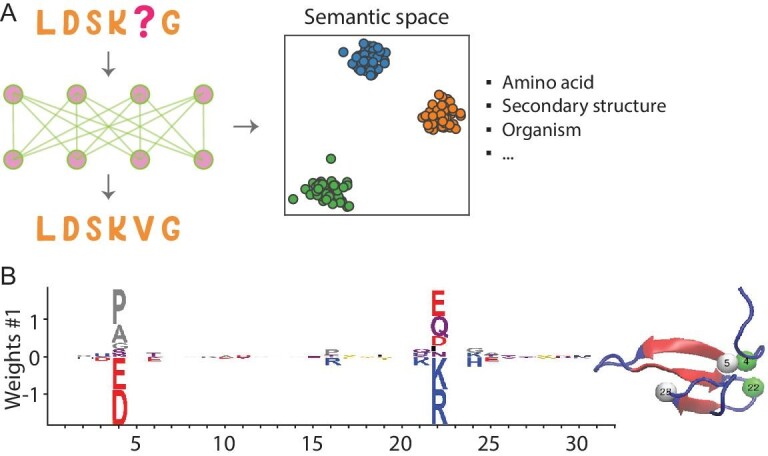
Deciphering protein sequence semantics with generative models. (A) Language models embody the semantics encoded in sequences, capturing various elements including amino acid types, secondary structure and information at the organism level, to name a few. (B) The weight logo associated with a hidden unit of the RBM model imparts biologically meaningful information. For example, the interactions of charged residues between residues 4 and 22 are underscored. This figure, adapted from ref. [75], is distributed under the terms of the Creative Commons Attribution License.

Language models can learn semantics by analyzing the co-occurrence patterns of amino acids in large volumes of sequence data, or by implicitly modeling the syntax and structure of protein language. The RNN model's hidden state was found to encode structural, evolutionary and functional information [45]. Remarkably, when projected into low-dimensional space, the embedding vectors for distinct amino acids arrange according to their physicochemical properties (Fig. 6A). The representation of protein sequences organizes based on the organism origin and protein secondary structure [45]. By carefully analyzing the correlation between neurons in the network and various properties, it is possible to comprehend the significance of different neurons. These observations extend to the transformer model as well [46].

Hie *et al.* also utilized the language framework to examine the semantics and fitness of viral proteins [72]. The language model's sequence embedding captures the semantics, where sequences in different semantic landscape areas may suggest antigenic differences. Furthermore, sequences with high probability in the language model indicate increased fitness. These two factors collectively influence the likelihood of a new viral protein mutation evading detection by the immune system.

Although language models have had great success in learning the semantics of natural language, their main focus has been on text-based inputs. However, protein sequences involve more than just text-based information, and also encompass evolutionary, biological and physical priors that traditional language models are unable to capture [18,73 ,74]. As a result, it is necessary to develop new language models that can incorporate these priors and go beyond semantic visualization.

Besides language models, using a restricted Boltzmann machine (RBM), a generative model akin to the MaxEnt model, Tubiana *et al.* managed to extract meaningful biological features from the learned representation [75]. Certain hidden units were assigned biological relevance, such as a hidden unit trained with the Kunitz Domain MSA that represented the interaction between charged residues at two separate sites (Fig. 6B). While it is unclear if each hidden unit can be assigned a definite biological meaning, this analysis offers valuable insights. This technique is somewhat related to predicting protein contacts using evolutionary information and protein sector analysis [41,63].

## APPLICATIONS IN ENZYME ENGINEERING

Generative models have been employed to design functional enzymes, as summarized in Table 2. For example, Russ *et al.* utilized DCA to design chorismate mutase homologs, resulting in 481 functional sequences from 1618 tested, an impressive success rate of ∼30% [76]. Biochemical assays characterized five designed sequences, demonstrating activity similar to their natural counterparts. Repecka *et al.*, using GANs, investigated malate dehydrogenase and found activity in 13 of 55 tested sequences [77]. Employing VAEs, Hawkins-Hooker *et al.* examined bacterial luciferase luxA, with 9 out of 11 tested sequences exhibiting activity [78]. Giessel, also utilizing VAEs, explored ornithine transcarbamylase [79]. Protein language models, in addition to models trained on MSA, have also been used to create lysozyme variants [80].

**Table 2.** Application of generative AI in enzyme engineering.

	**Generative**	**Mutant**	**Designs**	**Improved activity**
**Enzyme**	**model**	**type**	**functional?**	**vs. wild-type?**
Chorismate mutase	MaxEnt [76]	Multiple	✓	
Malate dehydrogenase	GAN [77]	Multiple	✓	
Luciferase LuxA	VAE [78]	Multiple	✓	
Lysozyme	Language [80]	Multiple	✓	
Ornithine transcarbamylase	VAE [79]	Multiple	✓	✓ (2.3-fold)
Luciferase RLuc	MaxEnt [64]	Single	✓	✓ (2.0-fold)

In most of these enzyme engineering endeavors, the tested sequences do not surpass wild-type or natural enzymes in performance, and even when improvements occur, numerous mutations have to be introduced. While such diverse variants with natural-like functions highlight the generative potential of machine-learning models, their practical application could face limitations due to factors such as increased cost, complexity, potential ethical concerns and poor solubility in some cases [81]. Hence, there is a need to further develop innovative methods to identify beneficial mutations, a task that falls within the realm of rational enzyme engineering.

Rational enzyme engineering demands a deeper understanding of enzyme action. As discussed in a previous section, the evolutionary information in various enzyme components is linked to distinct physicochemical properties, specifically, active sites and enzyme scaffolds are primarily shaped by the evolutionary pressure of enzyme activity and stability, respectively [60]. Recently, we applied this concept to RLuc luciferase [64]. The evolution-catalysis relationship was validated for RLuc using previous enzyme characterization data. We then followed the relationship to design mutants with potentially enhanced properties. Four out of eight single mutants surrounding the substrate exhibited improved activity compared to the wild-type, with minimal changes in stability. In contrast, three out of six expressed single mutants in the enzyme scaffold displayed increased stability but reduced activity. The overall experimental results confirm the evolution-catalysis relationship. Interestingly, the maximum fold enhancement in activity achieved through a single mutation in RLuc is 2.0, which is on par with the best generative design resulting from multiple mutations (on average eight mutations) in ornithine transcarbamylase, with a fold increase of 2.3 [79]. The results highlight the potential of using generative modeling for enzyme sequences in rational enzyme engineering.

## SUMMARY AND PERSPECTIVE

Generative models, which learn patterns underlying naturally evolved sequences, hold the potential to revolutionize our comprehension of enzymes and ultimately aid in designing efficient biocatalysts. In this review, we touched upon a few topics that focus on generative modeling of enzyme sequences. For a broader perspective on machine learning in protein studies, we recommend readers explore other reviews [12–26].

Implementing generative models to investigate enzyme physicochemical properties necessitates a large quantity of both sequencing data and enzyme biochemistry data. Fortunately, metagenomic databases, which house genetic material from microbial communities, are experiencing rapid growth [82]. Improvements in sequencing technologies could further boost data collection. However, the slow accumulation of physicochemical data for enzyme variants obstructs the establishment of relationships between sequences and functions. The standardization of enzymatic data reporting is crucial [83], and the application of microfluidics for high-throughput measurement of enzymatic data seems promising [43,84].

In addition to data, a crucial aspect of machine learning involves selecting a model that aligns with the inductive bias of a specific problem. Many existing models introduced here have been directly borrowed from other disciplines, which may not provide the optimal approach for addressing biological problems. This situation can hinder the ability to learn and extract genuine biological principles. From another perspective, it is worth considering the development of models specifically tailored to the unique requirements of biological research, including proposing biologically meaningful tokenization, structure-informed sequence embedding, and designing a good measure for sequence representation quality [18,74,85]. Furthermore, interdisciplinary collaboration between experts in enzymology, machine learning and physics-based models could foster the development of novel models that are better suited to biological problems and can effectively capture the inherent characteristics of biological systems.

While the focal point of our review is the application of generative modeling to enzyme sequences, we acknowledge the transformative potential of applying generative models to protein structures in a bid to enhance enzyme design. Recent breakthroughs in protein structure prediction have presented potent tools for translating protein sequences into structures [86], sparking the development of novel strategies that utilize generative models for *de novo* protein design. Grounded in the principles of non-equilibrium thermodynamics, generative diffusion models enable protein design under a variety of circumstances, including the scaffold of enzyme active sites [87,88]. However, the task of constructing efficient enzymes *de novo* remains challenging due to many mysteries surrounding enzyme catalysis [89]. As a result, physics-based models need to be improved and integrated with generative designs.

Additionally, natural language processing using generative language models, such as ChatGPT [30], can revolutionize our comprehension of enzymes. Specifically, ChatGPT can aid in extracting knowledge from previous enzyme research, expediting the discovery process. GPT-4, for example, envisions a wider range of applications for generative models in enzyme studies than our review outlines (see Supplementary Data). While some assertions might seem early, the prospects for upcoming advancements are bright.

In summary, the integration of generative models into enzyme studies holds the promise of expanding our knowledge of enzymology and facilitating the development of efficient enzymes for diverse applications, potentially giving rise to a novel domain called ‘evolutionary catalysis’. To this end, embedding domain expertise into generative models can enhance their capacity to address the specific complexities inherent in enzyme research and engineering.

## Supplementary Material

nwad331_Supplemental_File
